# SNP-SNP interactions between *WNT4* and *WNT5A* were associated with obesity related traits in Han Chinese Population

**DOI:** 10.1038/srep43939

**Published:** 2017-03-08

**Authors:** Shan-Shan Dong, Wei-Xin Hu, Tie-Lin Yang, Xiao-Feng Chen, Han Yan, Xiang-Ding Chen, Li-Jun Tan, Qing Tian, Hong-Wen Deng, Yan Guo

**Affiliations:** 1 Key Laboratory of Biomedical Information Engineering of Ministry of Education, School of Life Science and Technology, Xi’an Jiaotong University, Xi’an 710049, P. R. China; 2 Laboratory of Molecular and Statistical Genetics, College of Life Sciences, Hunan Normal University, Changsha 410081, P. R. China; 3 School of Public Health and Tropical Medicine, Tulane University, New Orleans, LA 70112, USA

## Abstract

Considering the biological roles of *WNT4* and *WNT5A* involved in adipogenesis, we aimed to investigate whether SNPs in *WNT4* and *WNT5A* contribute to obesity related traits in Han Chinese population. Targeted genomic sequence for *WNT4* and *WNT5A* was determined in 100 Han Chinese subjects and tag SNPs were selected. Both single SNP and SNP × SNP interaction association analyses with body mass index (BMI) were evaluated in the 100 subjects and another independent sample of 1,627 Han Chinese subjects. Meta-analyses were performed and multiple testing corrections were carried out using the Bonferroni method. Consistent with the Genetic Investigation of ANthropometric Traits (GIANT) dataset results, we didn’t detect significant association signals in single SNP association analyses. However, the interaction between rs2072920 and rs11918967, was associated with BMI after multiple testing corrections (combined *P* = 2.20 × 10^−4^). The signal was also significant in each contributing data set. SNP rs2072920 is located in the 3′-UTR of *WNT4* and SNP rs11918967 is located in the intron of *WNT5A*. Functional annotation results revealed that both SNPs might be involved in transcriptional regulation of gene expression. Our results suggest that a combined effect of SNPs via *WNT4*-*WNT5A* interaction may affect the variation of BMI in Han Chinese population.

Obesity is a complex medical condition that may lead to health problems, including heart disease, type 2 diabetes (T2D), and certain types of cancer^1^. Like many other complex diseases, obesity is the result of the combination of genetic susceptibility and environmental factors. Twin and family studies have shown that the heritability of body mass index (BMI) is 40–70%^2^,^3^, and other anthropometric measures of obesity have similar heritability^2^,^3^,^4^,^5^,^6^. Although genome-wide association studies (GWASs) have linked obesity with many genetic variants, known variants still account for only a small fraction of the heritability of obesity^7^. Therefore, more associated loci should be discovered.

The wingless-type MMTV integration site (WNT) signaling pathway plays important roles in regulating adipogenesis^8^. WNT molecules exert their effects through canonical WNT/β-catenin dependent or non-canonical WNT/β-catenin independent pathways. *In vivo* experiments have confirmed that both pathways are important in adipose tissue formation^9^. In rodents, inhibition of WNT10b of the canonical pathway could promote the differentiation of adipogenic precursor cells into mature adipocytes^10^. In humans, promotion of adipogenesis is related to the up-regulation of the Dickkopf-1, a known inhibitor of the canonical WNT signaling pathway^11^. *WNT5A* encodes a member of the WNT family. In mouse 3T3-L1 preadipocytes, Wnt5a is a positive regulator of adipogenesis at the beginning of adipocyte differentiation^12^. However, WNT5A signaling promotes human multipotent mesenchymal stem cells and human adipose tissue-derived mesenchymal stromal cells to undergo osteogenesis, while adipogenesis might be inhibited^13^,^14^. It is suggested that Wnt5a might inhibit adipogenesis through two mechanisms, suppressing the activity of *Ppar-γ* and enhancing the canonical WNT signaling through *Lrp5/6* expression^15^. Therefore, WNT5A might be an important switch molecule in regulating the osteoblastogenesis and adipogenesis of multipotent stem cells. Genetic variants in *Wnt5a* have been associated with obesity in mice models^16^. However, the associations between *WNT5A* variations and obesity related traits in human subjects are still unclear. *WNT4* encodes another WNT family member and it can also promote adipocyte differentiation in mouse 3T3-L1 preadipocytes at the initial stage of the differentiation^12^. In pancreatic islets of obese mice, WNT4 might inhibit the canonical WNT signaling^17^. A previous study suggested that a SNP in *WNT4* is a susceptibility locus for fat distribution in European ancestry individuals when combined with endometriosis^18^. However, the associations of *WNT4* polymorphisms with obesity related traits in subjects without special medical problems are still unknown.

It has been long known that genetic interactions can affect heritability calculations^19^. Generally, genetic interactions are not considered in SNP association analyses, leading to a substantial proportion of the missing heritability for complex diseases/traits^20^. Therefore, it is important and necessary to apply statistical methods to decipher genetic interactions and their relationships with disease susceptibility. Since *WNT4* and *WNT5A* are both involved in the noncanonical WNT pathway, gene-gene interactions may contribute to their roles in adipogenesis. Currently, whether the interaction between *WNT4* and *WNT5A* contributes to BMI variations is still unclear.

Since previous studies have implicated *WNT5A* and *WNT4* in adipogenesis, we hypothesized that *WNT5A* and *WNT4* might influence obesity related traits and might be candidate susceptibility genes for obesity. However, genetic variations contributing to their associations with obesity are still unclear. Therefore, in this study, we performed both single SNP and SNP × SNP interaction association analyses to investigate the effect of genetic variations of *WNT5A* and *WNT4* on BMI in Han Chinese subjects.

## Results

The basic characteristics of the subjects are listed in Table 1. The 100 unrelated subjects were sequenced successfully with the mean depth of at least 100.57 × and the coverage of target region for each sample was all over 92.5%. For *WNT4*, a total of 49 SNPs were identified (supplementary Table S1), with an average density of 1 SNP per 0.54 kb. 29 SNPs were identified for *WNT5A*, with the average density of 1 SNP per 0.76 kb (supplementary Table S1). The numbers of SNPs we identified were similar to the data from 1000 Genome phase III, which were 52 (*WNT4*) and 29 (*WNT5A*) in East Asian population, respectively. Using pairwise tagging with the r^2^ threshold of 0.8 in Haploview^21^, 10 and 8 tag SNPs were selected for *WNT4* and *WNT5A*, respectively (supplementary Table S2 and Table 2). Therefore, 18 single SNP analyses and 80 (10 × 8) SNP × SNP analyses were performed. The significance threshold after multiple testing correction was set as combined *P* < 5.10 × 10^−4^ (0.05/98).

### Single SNP association analyses

For the single SNP association analyses, no significant association results were obtained in the meta-analysis (*P* > 0.05, Table S3). In addition, we checked the association results of these tag SNPs in the Genetic Investigation of ANthropometric Traits (GIANT) dataset for BMI in all ancestries published in 2015^7^. Similarly, after multiple testing corrections, no significant association was detected.

### SNP × SNP interaction analyses

We carried out SNP × SNP interaction analyses between the two genes to explore the underlying mechanism. Meta-analyses results showed that the SNP pair rs2072920-rs11918967 was associated with BMI after multiple testing corrections (combined *P* = 2.20 × 10^−4^, Table 3). These two SNPs are located in the 3′-UTR of *WNT4* and intron4 of *WNT5A*, respectively. The interaction of rs2072920-rs11918967 was also significantly associated with BMI in each contributing data set, with the *P* values of 0.0122 in 100 Han Chinese and 0.0014 in 1,627 Han Chinese, respectively.

**Figure 1:**
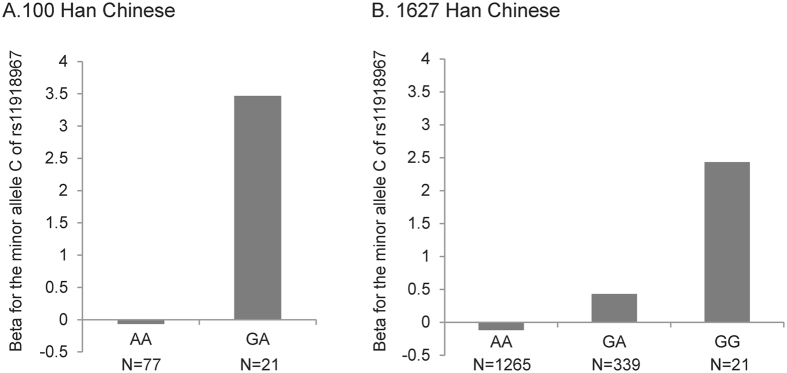
(**A**) Association of the minor allele “C” of rs11918967 with BMI in subjects carrying different genotypes of rs2072920 in the 100 Han Chinese subjects. There were only two subjects with “GG” of rs2072920, so we didn’t analyze the association results in this subgroup. (**B**) Association of the minor allele “C” of rs11918967 with BMI in subjects carrying different genotypes of rs2072920 in the 1,627 Han Chinese subjects. The beta values of the association analyses results are shown in the y-axis.

We further checked whether the effect of the minor allele “C” of rs11918967 on BMI was different between subjects carrying different genotypes of rs2072920 using the beta coefficient. As shown in Fig. 1A, in the 100 unrelated subjects, the minor allele “C” of rs11918967 was negatively associated with BMI in subjects carrying “AA” of rs2072920 (beta = −0.067, 95% CI = −1.653–1.518, standard error (se) = 0.809). However, it was positively associated with BMI in subjects carrying “GA” of rs2072920 (beta = 3.468, 95% CI = 1.454–5.481, se = 1.027). Similarly, in another sample of 1,627 Han Chinese subjects (Fig. 1B), the minor allele “C” of rs11918967 was also negatively associated with BMI in subjects carrying “AA” of rs2072920 (beta = −0.120, 95% CI = −0.350–0.110, se = 0.117). Consistently, it was positively associated with BMI in subjects carrying “GA” (beta = 0.430, 95% CI = −0.017–0.877, se = 0.228) and “GG” of rs2072920 (beta = 2.432, 95% CI = 0.797–4.068, se = 0.835). Therefore, the minor allele “C” of rs11918967 was positively associated with BMI in subjects with at least one copy “G” allele of rs2072920.

### Functional annotation

**Figure 2:**
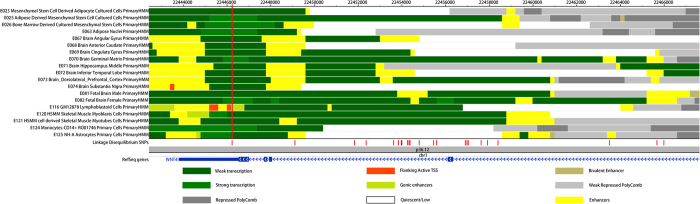
Annotation of rs2072920 in *WNT4* in tissues/cell lines that might be related to obesity. The longest vertical red line refers to rs2072920. SNPs in LD with rs2072920 were shown with short vertical red lines. Primary HMM refers to the chromatin states predicted by hidden Markov model based on combinations of histone modification marks.

We used information from tissues/cell lines that might be relevant to obesity (supplementary Table S4) to annotate the selected SNPs. As shown in Fig. 2, rs2072920 was located in the region of strong transcription in adipose derived mesenchymal stem cell cultured cells (AMSC), bone marrow derived cultured mesenchymal stem cells (BMSC), adipose nuclei, brain germinal matrix, fetal brain female and Monocytes-CD14 + cells. It was also located in the genic enhancer region of GM12878. Of note, rs58543510, which was in complete linkage disequilibrium (LD) with rs2072920 (r^2^ = 1, D’ = 1), was located in the enhancer region of 10 tissues/cell lines. Ten other LD SNPs were also found in the enhancer region of at least one tissue/cell line. RNA binding protein (RBP) data analyses showed that rs2072920 and its LD SNPs were located in the poly(A) binding protein cytoplasmic 1 (PABPC1) binding region. We further checked whether the enhancer SNPs affect transcription factor binding to known motifs. As shown in Table S5, 9 of the 10 enhancer SNPs fell within at least one critical position in transcription factor binding motifs. Specifically, the effect of rs58543510 on the T3R motif has been validated in various cell lines, including some cells that might be related to obesity, such as GM12878, skeletal muscle myoblasts cells (HSMM), and HSMM cell derived skeletal muscle myotubes cells (HSMMtube).

**Figure 3:**
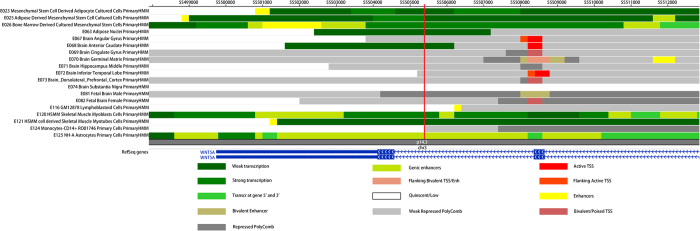
Annotation of rs11918967 in *WNT5A* in tissues or cell lines that might be related to obesity. The vertical red line refers to rs11918967. Primary HMM refers to the chromatin states predicted by hidden Markov model based on combinations of histone modification marks.

As shown in Fig. 3, rs11918967 was located in the region of strong transcription in AMSC, BMSC and HSMM. It was also located in the genic enhancer region of astrocytes. There were no SNPs in LD with rs11918967. RBP data analyses also showed that rs11918967 was located in the PABPC1 binding region. Motif analyses suggested that it might affect the binding motif of AP-2.

## Discussion

In this study, we aimed to investigate the genetic associations between *WNT4* and *WNT5A* polymorphisms and BMI in Han Chinese subjects. We performed meta-analyses using two independent samples including 100 and 1,627 Han Chinese subjects and the results showed that the interaction between rs2072920 in *WNT4* and rs11918967 in *WNT5A* was associated with BMI after multiple testing corrections. Our findings suggest that the interaction between *WNT4* and *WNT5A* contributes to BMI variations in Han Chinese population.

Although WNT5A is a factor inhibiting adipogenesis in humans^13^,^22^, the associations between *WNT5A* genetic variations and BMI have not been reported before. Both *WNT4* and *WNT5A* are known as noncanonical WNT genes^23^, and interaction between WNT4 and WNT5A protein has been proved by using high-throughput affinity-purification mass spectrometry^24^. Functional annotation analyses suggest that these two SNPs and their LD SNPs are located in strong transcription or enhancer region in at least one obesity related tissue/cell line. These regions could bind PABPC1, which is a poly(A) binding protein. Binding of PABPC1 to poly(A) tail of mRNA could promote translation initiation and it is also involved in the regulation of mRNA decay^25^. Since the SNPs we reported here are all located within or near the 3′-UTR regions, they may be involved in transcriptional regulation of gene expression through affecting the binding of PABPC1. Motif analyses results for SNPs in the enhancer region suggested that they may regulate gene expression through impacting the binding of transcription factors to known motifs. Further studies are needed to confirm the underlying mechanism of these SNPs in regulating gene expression.

We couldn’t detect any significant association results in single SNP association analyses in both *WNT4* and *WNT5A*. This is different from previous studies since a SNP in *WNT4* has been reported to be significantly associated with fat distribution in European ancestry individuals^18^. The inconsistence may be caused by the ethnic differences between European and Asian populations, since they have different LD structures and allele frequencies^26^. In addition, the association signal was detected in endometriosis patients^18^, which may lead to different results from our healthy subjects.

The detected interaction between rs2072920 and rs11918967 can explain 0.899% of the phenotypic variation. Given the sample size adopted, this study can achieve about 68.14% statistical power to detect the association signal that accounts for ~0.899% of the phenotypic variation. We acknowledge that this study is not powerful to detect association signals for variants with low effect size.

Limitations of the current study must be addressed. The two samples we used have notably different age and BMI distributions. We included age as covariate to adjust the BMI values, which could eliminate the effect of age to some extent. The SNP-SNP interaction association signals with *P* < 0.05 were also detected in each contributing data set, suggesting that the effect of rs2072920-rs11918967 interaction on BMI variations is independent from age and BMI distributions. We focused on the analyses in Han Chinese subjects and the results may not be applicable to other populations. Further studies are needed to investigate the association between *WNT4*-*WNT5A* interaction and BMI in other populations.

In summary, this study provides the evidence that the interactions between *WNT4* and *WNT5A* could affect the variation of BMI in Han Chinese subjects. Further investigations are needed to clarify our findings in other populations.

## Methods

### Ethics, consent and permissions

This study was approved by the Institutional Review Boards of Xi’an Jiaotong University. Signed informed consent was obtained from all subjects. All experiments were performed in accordance with relevant guidelines and regulations.

### Subjects

Detailed information of the subjects is described as follows:

#### Sample 1

100 unrelated healthy Han Chinese adults living in Xi’an and its neighboring areas were recruited. During physical examination of each individual, body weight and height were recorded. BMI was calculated as body weight (kg) divided by the square of height (m). Subjects with chronic diseases and conditions that affect fat metabolism were excluded. These disorders/conditions included diseases affecting vital organs (heart, lung, liver, kidney and brain) and severe endocrine, metabolic or nutritional diseases. The exclusion criteria were described in detail in previous studies^27^.

#### Sample 2

1,627 Han Chinese subjects were recruited from Xi’an and Changsha in Midwestern China. The exclusion criteria were the same as those used in the 100 unrelated subjects.

### Targeted gene sequencing for the sample 1

Targeted gene sequencing was performed in the 100 unrelated subjects. *WNT5A* and *WNT4* were provided to Roche NimbleGen, Inc. (Madison, WI, USA) for custom target region capture array design. Target region selection was accomplished by downloading the sequence and selecting the transcripts with the longest exons from the University of California Santa Cruz (UCSC) Genome Browser. These transcripts are NM_030761 for *WNT4* and NM_003392 for *WNT5A*. The array was designed to target the whole transcripts of the two genes and ± 1,000 bp flanking regions. DNA was extracted from whole blood using a commercial isolation kit (Gentra systems, Minneapolis, MN, USA). Qualified genomic DNA was randomly fragmented into fragments with a base pair peak of 100 to 200 bp. A pair of adapters was ligated to both ends of the fragments, which were then amplified, purified, and hybridized to the custom array for enrichment. The resulting DNA library was subjected to paired-end sequencing with read length of 90 bp on the Illumina HiSeq 2000 platform.

### Sequencing reads alignment and SNP detection in the sample 1

First, the adapter sequence in the raw data was removed, and nucleotides with a quality score lower than 20 were trimmed. The resulting filtered reads were mapped to the human reference genome (hg19) using the Burrows-Wheeler Aligner (version 0.7.10, command BWA-MEM)^28^. Sequence Alignment/Map (SAM) format alignment result files were imported to Samtools^29^ and the ‘rmdup’ command was used to remove potential PCR duplicates. SNPs were detected by SOAPsnp^8^ and annotated with ANNOVAR^30^. SNPs with minor allele frequencies (MAF) less than 0.05 and deviated from Hardy-Weinberg equilibrium (*P* < 0.001) were excluded. Haploview^21^ was used to select tag SNPs and only tag SNPs were used in the association analyses.

### Genotyping in the sample 2

For the 1,627 Han Chinese subjects, SNP genotyping was performed using Genome Wide Human SNP Array 6.0 (Affymetrix, Santa Clara, CA, USA), which has been detailed previously^31^. For SNPs which were not genotyped in the arrays, we imputed the genotypes with the IMPUTE^32^ to facilitate the comparison of association results. The 1000 Genome dataset was used as the reference data.

### Statistical analyses

BMI was adjusted for age and sex in a linear regression model. The resulting residuals were tested for normality by Kolmogorov-Smirnov test and the residuals of BMI in both sample sets were normally distributed. The above analyses were performed with the software MINITAB (Minitab Inc., State College, PA, USA). The residuals were then used in subsequent association analyses. At the single-marker level, association analyses for all SNPs assuming additive models of inheritance were carried out using PLINK^33^. In this model, the beta coefficient represents the rate of changes of the response variable as a function of the changes in the independent variable. Pairwise SNPs interactions were then tested by a linear regression analysis which was also performed with PLINK^33^. Briefly, PLINK makes a model based on allele dosage for each SNP, which fits a linear regression model in the following equation:

Y ~ β + β1*SNP1 + β2*SNP2 + β3*SNP1 × SNP2 + e

For “two copies” of A allele (minor allele) of SNP2 (SNP2 = 2), the equation is:

Y ~ (β + 2β2) + (β1 + 2β3) *SNP1 + e

For “one copy” of A allele of SNP2 (SNP2 = 1), the equation is:

Y ~ (β + β2) + (β1 + β3) *SNP1 + e

For “zero copy” of A allele of SNP2 (SNP2 = 0), the equation is:

Y ~ β + β1*SNP1 + e

Summary statistics of association analyses from the two samples were subjected to meta-analysis using the METAL software (http://csg.sph.umich.edu/abecasis/Metal/) under the sample-size weighted model. Multiple comparison problems were adjusted using the Bonferroni method.

We estimated the statistical power of our study using the Quanto v1.2.4 software (http://biostats.usc.edu/Quanto.html). The conservative significance threshold was set at *P* < 5.10 × 10^−4^.

### The Genetic Investigation of ANthropometric Traits (GIANT) dataset

The GIANT consortium is an international collaboration that aims to detect genetic loci associated with human anthropometric traits, including height and obesity related phenotypes. Summary statistics from large scale meta-analyses of genome wide single SNP association data are freely to access for all researchers. Here we downloaded the summary data for BMI from the article published in 2015^7^, which incorporated results from 322,154 European and 17,072 non-European-descent individuals (total *n* = 339,224). We used the results from all ancestries to validate our single-SNP association results.

### Functional annotation

In order to determine the potential regulatory function of SNPs associated with BMI, the SNPs were annotated with chromatin states predicted by hidden Markov model^34^ (HMM) based on combinations of histone modification marks, including H3K4me3, H3K4me1, H3K36me3, H3K27me3, and H3K9me3. The chromatin states data were obtained from the Roadmap project^35^. Detailed information of the states is shown in the Roadmap website (http://egg2.wustl.edu/roadmap/web_portal/chr_state_learning.html). Data from tissues or cell lines that might be relevant to obesity were obtained. Information for the cell lines or tissues we used is shown in supplementary Table S4. The annotation results were visualized in the WashU Epigenome Browser^36^.

We further used RNA binding protein (RBP) immunoprecipitation data from the ENCODE project^37^ to check whether the selected SNPs may affect gene expression through influencing protein binding. For the cell lines/tissues we selected for obesity (Table S4), only RBP data for GM12878 (B-lymphocyte, lymphoblastoid) are available now. The data were downloaded from the following URL: http://hgdownload.cse.ucsc.edu/goldenpath/hg19/encodeDCC/wgEncodeSunyAlbanyGeneSt/.

For SNPs in enhancer regions, we used HaploReg (v4.1)^38^ to check their effects on binding motifs. RegulomeDB^39^ was also used to check whether their effects on motifs binding were experimentally validated.

## Additional Information

**How to cite this article**: Dong, S.-S. *et al*. SNP-SNP interactions between *WNT4* and *WNT5A* were associated with obesity related traits in Han Chinese Population. *Sci. Rep.* **7**, 43939; doi: 10.1038/srep43939 (2017).

**Publisher's note:** Springer Nature remains neutral with regard to jurisdictional claims in published maps and institutional affiliations.

## Supplementary Material

Supplementary Tables

## Figures and Tables

**Table 1:** Basic characteristics of all subjects.

	Sample 1 (100 Han Chinese)			Sample 2 (1,627 Han Chinese)		
	Men (42)	Women (58)	Total (100)	Men (802)	Women (825)	Total (1,627)
Age (years)	49.67 ± 19.64	55.29 ± 11.82	52. 93 ± 15.75	31.43 ± 11.93	37.46 ± 13.77	34.49 ± 13.24
Height (cm)	171.52 ± 6.49	158.72 ± 6.10	164.10 ± 8.90	170.27 ± 5.96	158.38 ± 5.22	164.25 ± 8.16
Weight (kg)	74.36 ± 16.58	64.35 ± 11.58	68.56 ± 14.68	65.75 ± 9.64	54.63 ± 8.09	60.12 ± 10.48
BMI (kg/m^2^)	25.14 ± 4.63	25.55 ± 4.44	25.38 ± 4.50	22.66 ± 2.93	21.77 ± 3.05	22.21 ± 3.03

Data are shown as mean ± SD.

**Table 2:** Information of the tag SNPs.

Chromosome	Position	Rs#	Region	Gene
1	22444975	rs10737462	3′-UTR	*WNT4*
1	22446265	rs2072920	3′-UTR	*WNT4*
1	22449325	rs59709264	intron2	*WNT4*
1	22450487	rs2235529	intron2	*WNT4*
1	22451966	rs10917155	intron2	*WNT4*
1	22455717	rs77448785	intron2	*WNT4*
1	22455728	rs56673898	intron2	*WNT4*
1	22462609	rs12091003	intron1	*WNT4*
1	22463092	rs2865175	intron1	*WNT4*
1	22469069	rs60039305	intron1	*WNT4*
3	55499579	rs589557	downstream	*WNT5A*
3	55501002	rs669889	3′-UTR	*WNT5A*
3	55502251	rs3732750	3′-UTR	*WNT5A*
3	55505390	rs11918967	intron4	*WNT5A*
3	55508102	rs9818631	intron4	*WNT5A*
3	55509750	rs675575	intron3	*WNT5A*
3	55510008	rs12495121	intron3	*WNT5A*
3	55519857	rs648872	intron1	*WNT5A*

**Table 3:** Significantly associated SNP-SNP interactions in the two genes associated with BMI.

SNP1-SNP2	Combined *P*	100 Han Chinese subjects							1,627 Han Chinese subjects						
		Allele1	MAF1	Allele2	MAF2	Beta	se	*P*	Allele1	MAF1	Allele2	MAF2	Beta	se	*P*
rs2072920-rs11918967	2.20 × 10^−4^	G/A	0.125	C/G	0.285	3.5	0.0271	0.0122	G/A	0.1172	C/G	0.2983	0.7157	0.0065	0.0014

Note: Only significantly associated SNP pairs after multiple testing corrections are shown. se: standard error; Allele1: Alleles of SNP1; Allele2: Alleles of SNP2; MAF1: minor allele frequency of SNP1; MAF2: minor allele frequency of SNP2.
