# Supplementary material for: SNP-SNP interactions between WNT4 and WNT5A were associated with obesity related traits in Han Chinese Population
Source: Sci Rep. 2017 Mar 8;7:43939. doi: 10.1038/srep43939 (PMC5341019; doi:10.1038/srep43939)
Supplement: Supplementary Tables [file srep43939-s1.doc]

# SNP-SNP interactions between *WNT4* and *WNT5A* were associated with obesity related traits in Han Chinese Population

Shan-Shan Dong^1^, Wei-Xin Hu^1^, Tie-Lin Yang^1^, Xiao-Feng Chen^1^, Han Yan^1^, Xiang-Ding Chen^2^, Li-Jun Tan^2^, Qing Tian^3^, Hong-Wen Deng^3^, Yan Guo^1^*

1. Key Laboratory of Biomedical Information Engineering of Ministry of Education, School of Life Science and Technology, Xi'an Jiaotong University, Xi'an 710049, P. R. China
2. Laboratory of Molecular and Statistical Genetics, College of Life Sciences, Hunan Normal University, Changsha 410081, P. R. China
3. School of Public Health and Tropical Medicine, Tulane University, New Orleans, LA 70112, USA

**Running Title**: *WNT4*-*WNT5A* interaction affects obesity

**Corresponding Author**: Yan Guo, Ph.D.

Key Laboratory of Biomedical Information Engineering of Ministry of Education, and Institute of Molecular Genetics, School of Life Science and Technology, Xi'an Jiaotong University, Xi'an 710049, P. R. China

Tel: 86-29-82668463

Email: guoyan253@mail.xjtu.edu.cn

Table S1. SNPs identified for *WNT4* and *WNT5A* using target sequencing in the 100 unrelated samples.

| Chr_position | Rs# | Region | Gene | Minor | Major | MAF |
| --- | --- | --- | --- | --- | --- | --- |
| 1_22443760 | rs2235530 | downstream | *WNT4* | T | C | 0.205 |
| 1_22443887 | rs1046310 | 3’-UTR | *WNT4* | T | G | 0.135 |
| 1_22444975 | rs10737462 | 3’-UTR | *WNT4* | C | T | 0.27 |
| 1_22445991 | rs3765351 | 3’-UTR | *WNT4* | T | C | 0.15 |
| 1_22446265 | rs2072920 | 3’-UTR | *WNT4* | G | A | 0.125 |
| 1_22447316 | rs3765350 | intron4 | *WNT4* | A | G | 0.265 |
| 1_22449129 | rs58543510 | intron2 | *WNT4* | T | C | 0.125 |
| 1_22449239 | rs12037005 | intron2 | *WNT4* | T | C | 0.205 |
| 1_22449325 | rs59709264 | intron2 | *WNT4* | A | G | 0.145 |
| 1_22450487 | rs2235529 | intron2 | *WNT4* | C | T | 0.475 |
| 1_22451845 | rs7526484 | intron2 | *WNT4* | T | C | 0.125 |
| 1_22451966 | rs10917155 | intron2 | *WNT4* | A | G | 0.225 |
| 1_22452374 | rs11580864 | intron2 | *WNT4* | C | T | 0.125 |
| 1_22452714 | rs4655024 | intron2 | *WNT4* | T | C | 0.135 |
| 1_22453324 | rs10917157 | intron2 | *WNT4* | C | T | 0.22 |
| 1_22453626 | rs6676241 | intron2 | *WNT4* | C | T | 0.125 |
| 1_22453640 | rs2235528 | intron2 | *WNT4* | T | C | 0.225 |
| 1_22453842 | rs6678992 | intron2 | *WNT4* | C | T | 0.125 |
| 1_22453975 | rs2235527 | intron2 | *WNT4* | G | C | 0.125 |
| 1_22454001 | rs2235526 | intron2 | *WNT4* | C | T | 0.125 |
| 1_22454261 | rs6679479 | intron2 | *WNT4* | A | T | 0.125 |
| 1_22454325 | rs2235525 | intron2 | *WNT4* | A | G | 0.13 |
| 1_22454373 | rs10917158 | intron2 | *WNT4* | G | C | 0.125 |
| 1_22454791 | rs10917159 | intron2 | *WNT4* | A | C | 0.125 |
| 1_22455142 | rs7544210 | intron2 | *WNT4* | A | G | 0.13 |
| 1_22455449 | rs11582100 | intron2 | *WNT4* | G | A | 0.11 |
| 1_22455588 | rs11582542 | intron2 | *WNT4* | C | T | 0.125 |
| 1_22455717 | rs77448785 | intron2 | *WNT4* | T | C | 0.085 |
| 1_22455728 | rs56673898 | intron2 | *WNT4* | T | C | 0.135 |
| 1_22456895 | rs877629 | intron1 | *WNT4* | G | A | 0.125 |
| 1_22456970 | rs909816 | intron1 | *WNT4* | T | C | 0.125 |
| 1_22457027 | rs877628 | intron1 | *WNT4* | C | T | 0.125 |
| 1_22457611 | rs34087879 | intron1 | *WNT4* | A | G | 0.125 |
| 1_22457905 | rs78837735 | intron1 | *WNT4* | A | C | 0.125 |
| 1_22458384 | rs1076680 | intron1 | *WNT4* | T | C | 0.125 |
| 1_22458794 | rs12404660 | intron1 | *WNT4* | A | G | 0.255 |
| 1_22459170 | rs742358 | intron1 | *WNT4* | A | G | 0.13 |
| 1_22459754 | rs4655025 | intron1 | *WNT4* | G | A | 0.13 |
| 1_22460208 | rs10917161 | intron1 | *WNT4* | C | T | 0.225 |
| 1_22462111 | rs12037376 | intron1 | *WNT4* | G | A | 0.485 |
| 1_22462131 | rs10799737 | intron1 | *WNT4* | G | T | 0.465 |
| 1_22462609 | rs12091003 | intron1 | *WNT4* | A | C | 0.16 |
| 1_22463092 | rs2865175 | intron1 | *WNT4* | G | A | 0.085 |
| 1_22463457 | rs11805891 | intron1 | *WNT4* | G | T | 0.125 |
| 1_22465629 | rs57423947 | intron1 | *WNT4* | A | G | 0.125 |
| 1_22465820 | rs61768001 | intron1 | *WNT4* | T | C | 0.48 |
| 1_22465944 | rs59633390 | intron1 | *WNT4* | C | G | 0.125 |
| 1_22468215 | rs3820282 | intron1 | *WNT4* | C | T | 0.49 |
| 1_22469069 | rs60039305 | intron1 | *WNT4* | C | A | 0.125 |
| 3_55499407 | rs590386 | downstream | *WNT5A* | T | C | 0.065 |
| 3_55499579 | rs589557 | downstream | *WNT5A* | C | A | 0.055 |
| 3_55499664 | rs12497254 | downstream | *WNT5A* | G | A | 0.34 |
| 3_55499781 | rs1047898 | 3’-UTR | *WNT5A* | T | C | 0.345 |
| 3_55500408 | rs10865994 | 3’-UTR | *WNT5A* | T | A | 0.345 |
| 3_55501002 | rs669889 | 3’-UTR | *WNT5A* | G | C | 0.185 |
| 3_55501175 | rs1829556 | 3’-UTR | *WNT5A* | T | C | 0.34 |
| 3_55502251 | rs3732750 | 3’-UTR | *WNT5A* | A | G | 0.08 |
| 3_55503522 | rs3773606 | 3’-UTR | *WNT5A* | A | T | 0.065 |
| 3_55504730 | rs7622120 | intron4 | *WNT5A* | G | A | 0.335 |
| 3_55505023 | rs2682456 | intron4 | *WNT5A* | T | C | 0.345 |
| 3_55505390 | rs11918967 | intron4 | *WNT5A* | C | G | 0.285 |
| 3_55505730 | rs2682455 | intron4 | *WNT5A* | C | T | 0.345 |
| 3_55506138 | rs2682454 | intron4 | *WNT5A* | A | C | 0.345 |
| 3_55506490 | rs3773608 | intron4 | *WNT5A* | C | T | 0.06 |
| 3_55506567 | rs2056349 | intron4 | *WNT5A* | T | C | 0.19 |
| 3_55507281 | rs556874 | intron4 | *WNT5A* | C | T | 0.345 |
| 3_55508075 | rs3821658 | intron4 | *WNT5A* | T | C | 0.19 |
| 3_55508102 | rs9818631 | intron4 | *WNT5A* | A | G | 0.085 |
| 3_55509750 | rs675575 | intron3 | *WNT5A* | A | T | 0.335 |
| 3_55510008 | rs12495121 | intron3 | *WNT5A* | C | A | 0.055 |
| 3_55511675 | rs472631 | intron3 | *WNT5A* | A | G | 0.33 |
| 3_55514766 | rs73075443 | intron2 | *WNT5A* | T | A | 0.19 |
| 3_55516963 | rs815541 | intron1 | *WNT5A* | C | G | 0.055 |
| 3_55517047 | rs815540 | intron1 | *WNT5A* | G | A | 0.34 |
| 3_55519857 | rs648872 | intron1 | *WNT5A* | A | G | 0.055 |
| 3_55520027 | rs56031929 | intron1 | *WNT5A* | C | T | 0.19 |
| 3_55520778 | rs566926 | intron1 | *WNT5A* | G | T | 0.355 |
| 3_55521717 | rs620014 | upstream | *WNT5A* | A | C | 0.055 |

Table S2. Tag SNP selection results.

| Tag SNP | Alleles Captured |
| --- | --- |
| *WNT4* | |
| rs2072920 | rs2235525, rs11805891, rs1076680, rs877628, rs6679479, rs78837735, rs58543510, rs6676241, rs11580864, rs10917158, rs11582542, rs909816, rs2235527, rs2072920, rs57423947, rs34087879, rs2235526, rs59633390, rs10917159, rs7526484, rs6678992, rs877629, rs11582100 |
| rs10917155 | rs2235528, rs10917161, rs10917157, rs10917155, rs12037005, rs2235530 |
| rs56673898 | rs742358, rs56673898, rs4655025, rs7544210, rs4655024 |
| rs2235529 | rs61768001, rs10799737, rs2235529, rs12037376, rs3820282 |
| rs10737462 | rs12404660, rs10737462, rs3765350 |
| rs59709264 | rs1046310, rs3765351, rs59709264 |
| rs12091003 | rs12091003 |
| rs2865175 | rs2865175 |
| rs60039305 | rs60039305 |
| rs77448785 | rs77448785 |
| *WNT5A* | |
| rs675575 | rs1829556, rs2682456, rs2682454, rs1047898, rs12497254, rs10865994, rs7622120, rs815540, rs556874, rs472631, rs566926, rs2682455, rs675575 |
| rs589557 | rs589557, rs590386, rs3773606 |
| rs669889 | rs73075443, rs3821658, rs56031929, rs669889, rs2056349 |
| rs3732750 | rs3732750 |
| rs648872 | rs3773608, rs648872, rs815541, rs620014 |
| rs11918967 | rs11918967 |
| rs9818631 | rs9818631 |
| rs12495121 | rs12495121 |

Table S3. Meta-analysis results of the single SNP association analyses for the tag SNPs.

| Chr_Position | SNP | 100 Han Chinese *P* | 1627 Han Chinese *P* | Meta *P* |
| --- | --- | --- | --- | --- |
| 1_22444975 | rs10737462 | 0.5997 | 0.1477 | 0.1253 |
| 1_22446265 | rs2072920 | 0.7616 | 0.2288 | 0.2146 |
| 1_22449325 | rs59709264 | 0.6884 | 0.6945 | 0.6306 |
| 1_22450487 | rs2235529 | 0.3527 | 0.0358 | 0.0721 |
| 1_22451966 | rs10917155 | 0.0553 | 0.2205 | 0.4808 |
| 1_22455717 | rs77448785 | 0.7137 | 0.4894 | 0.5640 |
| 1_22455728 | rs56673898 | 0.5885 | 0.9164 | 0.8124 |
| 1_22462609 | rs12091003 | 0.1206 | 0.9461 | 0.6491 |
| 1_22463092 | rs2865175 | 0.4123 | 0.5102 | 0.3991 |
| 1_22469069 | rs60039305 | 0.7482 | 0.2102 | 0.1958 |
| 3_55499579 | rs589557 | 0.7962 | 0.1391 | 0.1716 |
| 3_55501002 | rs669889 | 0.5115 | 0.6846 | 0.8193 |
| 3_55502251 | rs3732750 | 0.2759 | 0.8307 | 0.6310 |
| 3_55505390 | rs11918967 | 0.1427 | 0.6526 | 0.4216 |
| 3_55508102 | rs9818631 | 0.1234 | 0.2224 | 0.1169 |
| 3_55509750 | rs675575 | 0.1769 | 0.3306 | 0.2003 |
| 3_55510008 | rs12495121 | 0.4189 | - | - |
| 3_55519857 | rs648872 | 0.8712 | 0.2786 | 0.3134 |

Table S4. Tissue or cell types used to obtain chromatin state information for SNP annotation

| ID | Abbreviation | Description |
| --- | --- | --- |
| E023 | FAT.MSC.DR.ADIP | Mesenchymal Stem Cell Derived Adipocyte Cultured Cells |
| E025 | FAT.ADIP.DR.MSC | Adipose Derived Mesenchymal Stem Cell Cultured Cells |
| E026 | STRM.MRW.MSC | Bone Marrow Derived Cultured Mesenchymal Stem Cells |
| E063 | FAT.ADIP.NUC | Adipose Nuclei |
| E067 | BRN.ANG.GYR | Brain Angular Gyrus |
| E068 | BRN.ANT.CAUD | Brain Anterior Caudate |
| E069 | BRN.CING.GYR | Brain Cingulate Gyrus |
| E070 | BRN.GRM.MTRX | Brain Germinal Matrix |
| E071 | BRN.HIPP.MID | Brain Hippocampus Middle |
| E072 | BRN.INF.TMP | Brain Inferior Temporal Lobe |
| E073 | BRN.DL.PRFRNTL.CRTX | Brain Dorsolateral Prefrontal Cortex |
| E074 | BRN.SUB.NIG | Brain Substantia Nigra |
| E081 | BRN.FET.M | Fetal Brain Male |
| E082 | BRN.FET.F | Fetal Brain Female |
| E116 | GM12878 | Lymphoblastoid Cells |
| E120 | HSMM | Skeletal Muscle Myoblasts Cells |
| E121 | HSMMtube | HSMM cell derived Skeletal Muscle Myotubes Cells |
| E124 | Monocytes-CD14+ | Monocytes-CD14+ RO01746 Primary Cells |
| E125 | NH-A | Astrocytes |

Table S5. Motif analyses results for the enhancer SNPs

| Enhancer SNPs | Motifs changed | Experimentally supported cell/tissues |
| --- | --- | --- |
| rs2072920 | AP-4; TAL1 | - |
| rs58543510 | T3R | 8988T; Chorion; CLL; Fibroblast; Glioblastoma; GM12878; H1hesc; HeLa-S3; Hepatocytes; Hepg2; HMEC; HSMM; HSMMtube; Htr8; Huh75; LNCaP; MCF7; Melanoma; Myometrium; Pancreatic islets; pHTE |
|  | FOXP3 | - |
| rs6676241 | TBX5 | 293T |
|  | E2A | - |
| rs6678992 | RXRA | - |
| rs2235527 | E2A; NF-E2; SMC3; TAL1 | - |
| rs2235526 | ER | Hepatocytes |
|  | HNF4 | - |
| rs6679479 | aMEF-2 | GM12892; Huh7 |
|  | CEBPG | - |
| rs2235525 | ATF3; BDP1; SIX5; TBX5; P300 | - |
| rs10917158 | PLAG1; PPAR; RREB; SREBP | - |
| rs10917159 | c-Ets-1 | H1hesc |
| rs11805891 | - | - |
| rs57423947 | SMAD4 | - |
| rs11918967 | AP-2 | - |

Note: 8988T: pancreas adenocarcinoma; CLL: chronic lymphocytic leukemia cell; GM12878: B-lymphocyte, lymphoblastoid; H1hesc: embryonic stem cells; Hepg2: hepatocellular carcinoma; HMEC: mammary epithelial cells; HSMM: skeletal muscle myoblasts; HSMMtube: skeletal muscle myotubes differentiated from the HSMM cell line; Htr8: trophoblast cell line; Huh75: hepatocellular carcinoma; Huh7: hepatocellular carcinoma; LNCaP: prostate adenocarcinoma; MCF7: mammary gland adenocarcinoma; pHTE: primary tracheal epithelial cells; 293T: Human embryonic kidney cells 293
